# Clinical progress note: Hepatitis A virus

**DOI:** 10.1002/jhm.70171

**Published:** 2025-09-11

**Authors:** James M. McCluskey, Robyn A. Bockrath, Ravi Jhaveri

**Affiliations:** ^1^ Division of Pediatric Infectious Diseases Ann & Robert H. Lurie Children's Hospital of Chicago Chicago Illinois USA; ^2^ Northwestern University Feinberg School of Medicine Chicago Illinois USA; ^3^ Pediatric Hospital Based Medicine Ann & Robert H. Lurie Children's Hospital of Chicago Chicago Illinois USA

## Abstract

Despite a decline in hepatitis A virus (HAV) incidence following vaccine introduction, HAV remains a public health concern in the United States. Recent multi‐state outbreaks have demonstrated that HAV can re‐emerge and highlight inequities in vaccine coverage or access, outbreak response, and in those with social or health risk factors. Continued investment in prevention, particularly vaccination and surveillance, is essential to prevent resurgence. Hospitalization offers a critical opportunity to address these gaps and provide equitable protection for at‐risk populations.

## Visual Abstract

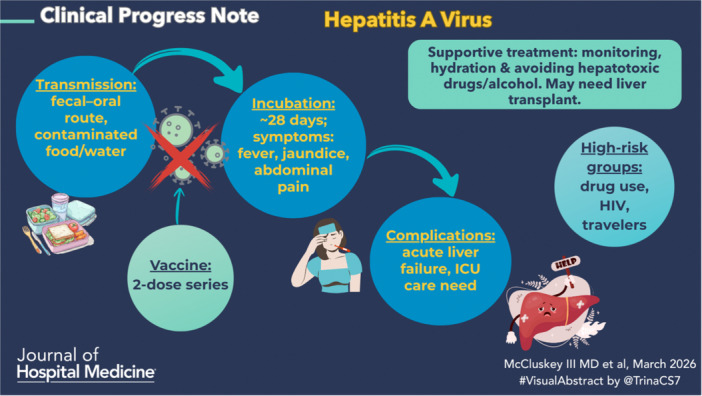

## INTRODUCTION

Hepatitis A virus (HAV) is a single‐stranded, linear ribonucleic acid (RNA) virus that was first identified in 1973.^1^, ^2^ Since that time, HAV has been classified as a member of the hepatovirus genus and within the *Picornaviridae* family. HAV is transmitted primarily through the fecal/oral route, shedding from the stool of infected individuals as naked, non‐enveloped virions.^1^ An individual can then become newly infected by ingesting contaminated food, water, or by direct contact with an infected person. HAV replicates in the gut mucosa, and after an average incubation period of 28 days (range 5–50 days), it eventually penetrates the gut.^3^ Cloaked in a membrane derived from these original host cells, it travels via the portal blood to the liver at which point symptoms can develop.^4^

## SEARCH STRATEGY

The authors reviewed the recently updated HAV guidance from the Centers for Disease Control and Prevention (CDC).^5^ Further literature review using PubMed was performed using keywords “hepatitis A,” “outbreak,” “vaccination,” and “hospitalization.”

## HEPATITIS A VACCINE AND RECOMMENDED SCHEDULE

Key lessons learned from recent outbreaks have included proactive control strategies, especially vaccination, among high‐risk groups before or in early stages of outbreaks.^6^ Thus, in response to the recent outbreaks, for those who did not already receive vaccine as a child, the 2020 Advisory Committee on Immunization Practices (ACIP) recommendations expanded to include injection and non‐injection drug use as well as housing insecurity as vaccine indications.^5^ These individuals are considered to be at high risk of acquiring HAV, as are men who have sex with men (MSM), those working with HAV in research settings, travel to countries endemic with HAV, and close contact with international adoptees in the first 60 days after arrival from a country endemic with HAV. Those indicated to receive HAV vaccine due to risk of severe disease further include those with chronic liver disease, human immunodeficiency virus (HIV) infection, and pregnancy.^7^ Lastly, the vaccine is also indicated for any person who requests vaccination.

The notable contraindication to HAV vaccination is severe allergy to any component of the vaccine, but side effects are also an important consideration. When the CDC reviewed the Vaccine Adverse Event Reporting System (VAERS) from 2006 to 2018, the most frequently reported adverse events were fever (13.6%), headache (11.5%), pain (11.5%), injection site pain (9.8%), and dizziness (9.4%) (CDC, unpublished data, 2006–2018). However, it should be noted that most of these adverse events were noted after visits with multiple vaccines being administered.^5^

HAV vaccine should be offered as an intramuscular (IM) two‐dose series starting at 12 months of age, spaced by 6 months, and for adults, the two‐dose series can be offered as well. Formulations approved for adult and pediatric patients have different dosing for these age groups, and for those greater than 18 years of age, it may be preferable to use the three‐dose combined HAV and hepatitis B (HBV) vaccine for dual protection as long as the indication is not for postexposure prophylaxis (PEP).^5^, ^7^ Further, preexposure prophylaxis (PrEP) and PEP may be considered. These are also approved uses of the vaccine given the rapid protective antibody production following vaccination (>91% within a month of the first dose).^5^ Also, if a subsequent dose is given, the upper limits of protective antibody production within a month of the second dose is 100%.^5^ Although the details are beyond the scope of this article, generally PrEP is considered for those traveling to endemic areas and may consist of vaccine and/or IM immunoglobulin (IG) depending on age and risk category. Combined HAV/HBV vaccine is typically three doses administered at 0, 1, and 6 months, however when using for PrEP, an accelerated schedule can be offered at 0, 7, and 21–30 days, followed by a booster at 12 months.^5^ Meanwhile, PEP is considered for those who have been exposed to HAV within the last 2 weeks, such as through sexual or household contact, or contaminated food.^5^ PEP consists of immunization for those at standard risk who have not already received vaccine, and IG administration for those that are ineligible for vaccine and/or at especially high risk (e.g., those <1 year of age or ≥40 years of age).^5^

## VACCINE IMPACT

The three HAV vaccine formulations available in the United States are inactivated and antigen based, and their impact has fundamentally changed the epidemiology of HAV in the United States following the first being licensed and implemented in 1995.^5^ From 1980 to 1995, leading into the vaccine approval, approximately 22,000 to 36,000 HAV cases were reported annually to the CDC, with 11% to 22% requiring hospitalization.^8^ However, one modeling analysis estimated an average of 271,000 actual infections per year from 1980 to 1999.^9^ Of these, an estimated 100 persons died of acute liver failure yearly due to HAV.^8^

Incremental steps were taken to construct the current vaccine schedule recommendations after the Food and Drug Administration (FDA) approval of the first HAV vaccine in 1995. In 1996, ACIP recommended vaccination of children aged ≥2 years who lived in areas with high HAV infection rates, persons of any age at increased risk of acquiring HAV or sequelae of infection, and in outbreaks.^5^ In 1999, ACIP recommendations expanded to include vaccination for children aged ≥2 years in states with average incidence rates that were twice the national average from 1987 to 1997 (i.e., ≥20 cases per 100,000 population) and consideration of vaccination for this age group in states where the incidence rate was at all greater than the national average.^5^ Finally, in 2006, ACIP recommendations expanded down to 12 months of age, regardless of incidence rate, matching those we have today.^5^, ^7^ During the implementation of these vaccine recommendations, from 1996 to 2011, the United States experienced a 95.5% decrease in reported HAV cases.^5^

The primary source of sporadic increases in HAV incidence have been attributed to foodborne outbreaks. Beginning in 2016, an increase of outbreaks involved 37 states, approximately 44,650 cases, 27,250 hospitalizations, and 415 deaths as of 2022.^10^ This cluster of outbreaks has been attributed to person‐to‐person transmission among individuals that used drugs and/or were experiencing homelessness, signaling a shift in US HAV epidemiology from point‐source food‐related outbreaks to larger community outbreaks.^10^

### Clinical manifestations and complications

HAV infection may have an abrupt and nonspecific symptom onset, including fever, malaise, anorexia, nausea, abdominal discomfort, dark urine, clay‐colored stools, and jaundice. In all, these clinical manifestations may be indistinguishable from hepatitis caused by other viruses.^1^ The likelihood of having symptoms is related to age with about 70% of infections in children <6 years of age being asymptomatic.^8^, ^11^ Among older children and adults, infection is symptomatic with jaundice in >70% of individuals.^8^ Young children were suspected to have been a key source of transmission before vaccination was available and recommended, as the majority of infected young children were asymptomatic or unrecognized while shedding HAV in their stool for months.^5^

While HAV infection is traditionally described as a self‐limited disease in healthy hosts, resolving in 2–3 months but sometimes lasting up to 6 months, recent outbreaks have highlighted significant complications, morbidity, and mortality.^6^, ^12^ Disease severity is known to be higher in those with older age, immunocompromise, chronic liver disease, or in those that have other underlying health conditions.^5^ Historically, reported liver failure occurred in <1% of cases, but has been as high as 4.3% in more recent outbreaks.^5^, ^6^ Other recently reported complications included acute liver injury (up to 80.4% of cases), need for liver transplantation (up to 0.6%), hepatic encephalopathy (up to 7%), and sepsis (up to 43%). Other less commonly reported complications include acute kidney injury, ascites, acute‐on‐chronic liver failure, fulminant, cholestatic, and relapsing hepatitis, malnutrition, decompensated cirrhosis, and renal or respiratory failure.^6^

These recent outbreaks demonstrate a significant medical and economic burden on hospital systems with hospitalization rates from 41.6% to 84.4% and intensive care unit (ICU) utilization ranging from 6.3% to 26.5%. Of those hospitalized, the mean length of stay was 2.5–6 days with a 30‐day readmission rate ranging from 4.2% to 23.4%.^6^ Financially, the average cost per HAV‐related hospitalization in the United States was estimated at $16,232 in 2017.^13^

### Diagnosis

**Figure 1:**
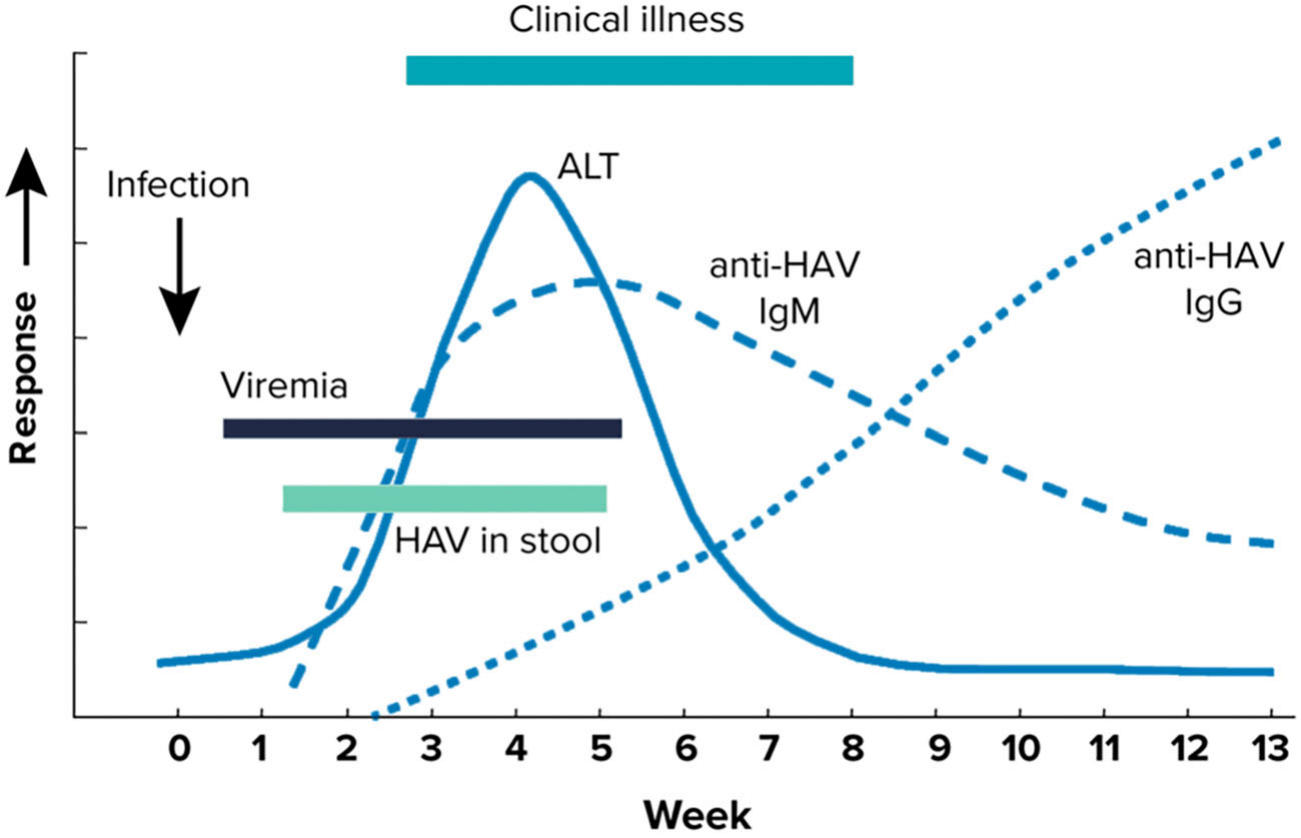
Typical serologic course of HAV infection and recovery. *Source*: Centers for Disease Control and Prevention. Available at: https://www.cdc.gov/hepatitis-a/media/images/2024/04/hepatitis-a-clinical-testing-guidelines.jpg. ALT, alanine aminotransferase; HAV, hepatitis A virus, Ig, immunoglobulin.

The earliest biochemical evidence of hepatitis includes elevated aspartate aminotransferase (AST), alanine aminotransferase (ALT), and serum bilirubin. These increases usually occur 5 to 10 days before symptom onset (Figure 1).^12^ HAV infection is then most commonly diagnosed by serologic testing, specifically, detection of immunoglobulin M (IgM) anti‐HAV, which usually appears within 5 to 10 days of symptom onset, and persists for about 4 months.^5^, ^12^ Immunoglobulin G (IgG) antibody titers rise later and persist for years. HAV IgM can be ordered as a standalone test and is often included in acute hepatitis test panels along with HBV surface antigen, HBV core antibody, and Hepatitis C antibody. For hospital‐based clinicians, proficiency in HAV diagnostic testing is highly relevant, with the majority of diagnoses made in the inpatient (84.4%) versus outpatient (15.6%) setting.^14^

## TREATMENT

Treatment of HAV infection is mainly supportive, including clinical and laboratory monitoring, with avoidance of hepatotoxic drugs and alcohol.^1^ In instances of life‐threatening and/or fulminant liver failure, transfer to a facility where liver transplantation can be performed should be considered.

Given the financial burden and strain on hospital systems, the primary management of the disease is in the prevention of infection acquisition and spread to others. In the hospital setting, contact precautions should be used when caring for patients with HAV infection who are diapered or incontinent, while standard precautions for all others is appropriate with optimal hand hygiene, as well as cleaning and disinfection of the environment.^5^

Finally, it would be prudent for hospitalists, as well as consulting gastroenterologists and infectious disease specialists, to assess immune status by reviewing vaccine records and/or checking HAV IgG titer to see if a patient should be offered a HAV vaccine before hospital discharge. Although no formal society recommendation exists on this, recent data revealed that <20% of patients with chronic liver disease have received any HAV vaccine, and hospitalization remains an opportune time to provide preventative measures.^15^

## CONCLUSION

HAV remains a public health concern despite the success of the vaccination in reducing HAV incidence in the United States. Recent outbreaks in the United States, particularly among those who use drugs or experience homelessness, underscore the importance of maintaining vaccination efforts and identifying HAV cases to inform ongoing infection control efforts.

## CONFLICT OF INTEREST STATEMENT

The authors declare no conflicts of Interest.
